# Mucus, commensals, and the immune system

**DOI:** 10.1080/19490976.2022.2041342

**Published:** 2022-03-03

**Authors:** Qing Zhao, Craig L. Maynard

**Affiliations:** a Department of Medicine, University of Alabama at Birmingham, Birmingham, 35294, USA; b Department of Pathology, University of Alabama at Birmingham, Birmingham, 35294, USA

**Keywords:** Colon mucus layer, mucus-associated bacteria, anti-commensal IgA, anti-commensal IgG, T-dependent, T-independent, flagellin, lachnospiraceae

## Abstract

The immune system in the large intestine is separated from commensal microbes and comparatively rare enteric pathogens by a monolayer of diverse epithelial cells overlaid with a compact and adherent inner mucus layer and a looser outer mucus layer. Microorganisms, collectively referred to as the mucus-associated (MA) microbiota, physically inhabit this mucus barrier, resulting in a dynamic and incessant dialog to maintain both spatial segregation and immune tolerance. Recent major findings reveal novel features of the crosstalk between the immune system and mucus-associated bacteria in health and disease, as well as disease-related peripheral immune signatures indicative of host responses to these organisms. In this brief review, we integrate these novel observations into our overall understanding of host-microbiota mutualism at the colonic mucosal border and speculate on the significance of this emerging knowledge for our understanding of the prevention, development, and progression of chronic intestinal inflammation.

## Introduction

Symbiosis between the host and the intestinal microbiota is dictated by the physical location of these organisms along the GI tract, including their proximity to the gut epithelium. The mammalian host has evolved multiple mechanisms that actively limit the direct interaction of gut commensals with host epithelial cells and immune cells in the underlying lamina propria. These include the epithelium-derived immunomodulatory and anti-microbial peptides, and the secretion of dimers from broadly reactive immunoglobulin A (IgA), that can help to sequester microbes away from the single-cell epithelial layer.^1,2^ In addition, specialized epithelial cells known as goblet cells (GC), which represent approximately 10–30% of human colonic epithelial cells, produce the secreted mucin, mucin-2 (Muc2) which forms a dense hydrogel or mucus layer that adheres to the apical surface of colonic epithelial cells. Continuous foraging of the outer region of the mucus layer by mucolytic bacteria helps to produce a loose outer mucus layer that serves as a permanent habitat for commensals including non-mucolytic opportunists such as *Escherichia coli* (*E. coli*).^3^ Previously, the term loosely adherent is used to refer to those organisms that forage the luminal edge of the outer mucus layer and, in so doing, supports their own existence while defending against pathogen colonization. Some commensals are also capable of occupying the dense inner mucus layer directly overlaying the epithelium. Thus, the gut microbiota is often thought of as luminal versus mucus-associated with the mucus-associated further divided into inner and outer mucus communities. Metagenomic profiling of almost 400 human gut microbiomes found that almost 90% of the organisms encode enzymes capable of cleaving or catabolizing mucin *O*-glycan monosaccharides suggesting that, in theory, most commensal organisms are capable of surviving in close association with the mucus layer.^4^ This is consistent with the fact that mucus-associated and luminal organisms are not phylogenetically distinct and taxonomic representation is virtually identical as both communities constantly admix.^5^ Notably, however, for the same species, there are functional differences reflected in the transcriptional profiles of bacterial cells of the same species recovered from the lumen, mucus layer, or physically associated with the gut epithelium.^6,7^ Thus, the term mucus-associated is a spatio-temporal description that also encompasses distinct functional and metabolic activities and immune system interactions that enable persistence in that niche. The relative proximity of these mucus-associated organisms to the epithelial border at any point in time arguably increases the likelihood of crosstalk with the intestinal immune system, from epithelial cells to the immune cells in the underlying lamina propria. Accordingly, the chronic diseases in the gastrointestinal tract collectively referred to as inflammatory bowel disease (IBD) are characterized by reproducible changes in the abundance of specific bacteria known to inhabit the mucus layer. Moreover, seroreactivity of antigens derived from these ‘border-dwellers’ is actively utilized in IBD diagnostics. Here, we discuss recent findings at the intersection of the gut mucosal barrier, the mucus-associated microbiota, and the intestinal immune system in homeostasis and in IBD. We posit that recent notable advances implore future exploration of host–microbiota interactions in the intestines that involve functional analyses of communities isolated directly from the mucus layer, elucidation of the immune cascades that are central to the persistence of these organisms in this locale, and determination of the possible connection(s) between these mutualistic interactions and the robust disease-specific anti-commensal immunoreactivity detectable systemically in patients with IBD.

### Production of the colonic mucus layer

**Figure 1.**
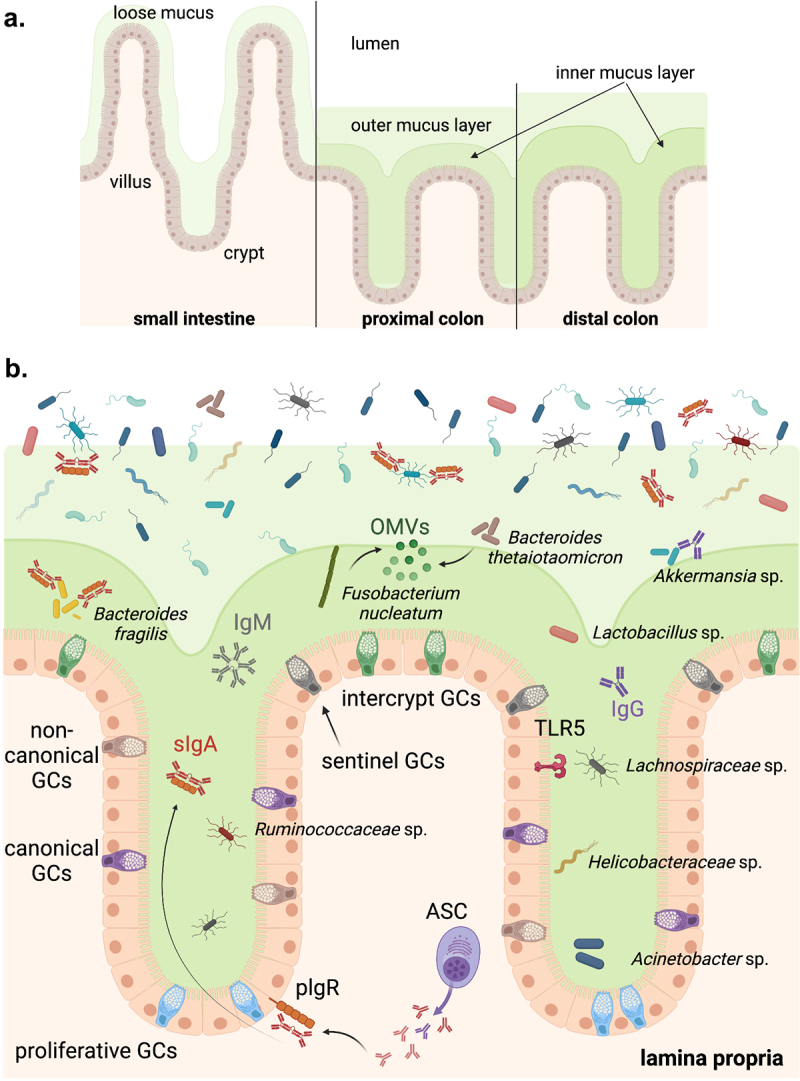
Host-microbiota spatial dynamics at the gut epithelial border. (A) The small intestinal epithelium is covered with a loose mucus layer, whereas a dense inner mucus layer and a looser outer mucus layer overlay the colonic epithelium. Both the density and thickness of the inner mucus layer increases from the proximal to the distal colon. (B) The colonic epithelial layer includes multiple subtypes of mucus-secreting goblet cells (GC). Crypt GCs include canonical and non-canonical GCs, as well as proliferative GCs that locate at the base of the crypts, whereas intercrypt GCs are found on the surface epithelium. TLR5, the receptor for bacterial flagellin, is constitutively and uniquely expressed on the luminal surface of colonic epithelial cells in the proximal colon. Microbial density is significantly higher in the outer compared to the inner mucus layer and the crypts. Bacteria including *Bacteroides thetaiotaomicron* and *Fusobacterium nucleatum* can bind mucin and release outer membrane vesicles (OMVs) which can lead to immune activation. Flagellated bacteria belonging to the *Lachnospiraceae, Ruminococcaceae*, and *Helicobacteraceae* families can colonize the crypts of the proximal colon. In addition to the physical barriers of the epithelial and mucus layers, lamina propria plasma cells produce anti-commensal immunoglobulins (Ig) including IgM, IgA, and IgG which help to regulate bacterial activation of the immune system. The most widely studied, IgA is transported into the lumen via the polymeric Ig receptor (PIGR) and helps to sequester bacteria and bacterial antigens away from the gut epithelium, but also facilitates colonization by bacteria such as *Bacteroides fragilis.*

Unlike the small intestine, which features a single unattached mucus layer, the colonic mucus comprises a dense, adherent inner mucus layer and a looser, more hydrated outer layer (Figure 1A). Membrane and secreted mucins appear to have been involved in the evolutionary compromise underlying host-microbiota mutualism, particularly in the densely populated large intestine. Mucins are integral to the persistence of commensals and the resistance to colonization by pathogenic competitors. Accordingly, despite the utilization of mucus for nutritional purposes, one microbiota adaptation that helps to limit the requirement for gut mucins as a food source is the collective expression of multiple enzymes, not encoded by mammals, that can catalyze the fermentation of complex dietary plant polysaccharides into short-chain fatty acids.^8^ Secreted mucins are produced by goblet cells (GCs) and are released apically into the intestinal lumen via a process regulated by the Forkhead box protein O1 (Foxo1).^9^ These mucins form a dense disulfide-bonded hydrogel – the mucus layer – overlying the epithelial cells. In the colon, the mucus layer is highly dynamic with the main mucus protein components displaying a turnover rate approximately twice that of epithelial cells in the same region of the intestine independent of the microbiota.^10^

GCs are highly specialized epithelial cells that, in addition to mucus production and secretion, are also essential for microbial antigen translocation, and the establishment of immune homeostasis during early life.^11^ An elegant study by Nystrom et al. demonstrated that based on their transcriptomic and proteomic profiles, intestinal GCs can be segregated into three major subsets.^12^ Proliferative GCs are found in the lower regions of intestinal crypts and give rise to two additional GC subsets – canonical GCs featuring an expression profile resembling that of mature GCs and non-canonical GCs with a transcriptional signature more closely overlapping than that of non-GC epithelial lineages (Figure 1B). Canonical and non-canonical GCs lining the intestinal crypts (collectively referred to as crypt GCs) secrete a thick, highly impenetrable mucus plume that fills and overlays the crypts, helping to protect the associated epithelial renewal machinery from bacterial invasion. Non-canonical crypt GC also give rise to sentinel GC that protect the crypt openings.^13^ In contrast, the terminal population of the canonical GC trajectory are the fully differentiated GCs of the surface epithelium or that open into the lumen and are collectively referred to as intercrypt goblet cells (icGCs). The icGCs produce a mucus layer that, despite being more permeable than the crypt mucus, helps to protect the surface epithelium from bacterial invasion. In both mice and humans, disruption of this intercrypt mucus results in microbiota-driven colonic inflammation.^12^

### Major constituents of the colonic mucus layer

The primary component of the secreted colonic mucus layer is Muc2, which is heavily glycosylated. In fact, approximately 80% of the total molecular weight of mucins is derived from *O*-glycan carbohydrates.^14^ The non-redundant role of Muc2 in the structure and integrity of the colonic mucus barrier is evident based on the severe pathology that occurs in the distal colons of Muc2-deficient mice.^15–18^ Relative to that of the distal colon, the inner layer of the proximal colon displays a slightly reduced density and correspondingly enhanced permeability,^19^ which likely has important ramifications for host-microbiota crosstalk in the proximal colon. In addition to the secreted mucus layer, transmembrane mucins are tethered to the apical surface of gut epithelial cells by a single transmembrane domain and perform structural and anti-microbial functions (reviewed in ^20^). Like Muc2, the extracellular domains of these mucins are heavily glycosylated and can be released from the cell upon interacting with bacteria, representing an additional mechanism for restricting microbial access to epithelial cells.

Determination of mucus-associated proteins can be challenging since mucus recovered from tissue in experimental or clinical settings can potentially contain substances in transit through the intestinal lumen or derived from dead epithelial or other cells.^10^ An earlier study identified over 1000 proteins that were considered mucus associated, among them, several immune related proteins including anti-microbial peptides and various components of immunoglobulins.^21^ A recent, more refined analysis of sigmoid colon biopsies from healthy individuals and ulcerative colitis patients proposed a core mucus proteome of 29 proteins.^22^ These include Muc2, chloride channel regulator, calcium-activated-1 (Clca1), and zymogen granulae protein 16 (Zg16), as well as IgA and proteins associated with its dimerization and transport, J-chain (IGJ) and polymeric IgA receptor (PIGR), respectively. The proteome also includes the Fc fragment of IgG binding protein (FCGBP), the abundance and distribution of which is similar to that of MUC2. FCGBP is expressed in mucus secreting cells in the intestines and can bind covalently to MUC2 and other mucus proteins in both humans and mice via cleaved von Willebrand D domains.^21,23^ In mice, expression of FCGBP is sparse in the mucus layers of the stomach and small intestine and the most robust and consistent expression has been observed in the proximal colon followed by the distal colon.^24^ The name derives from studies demonstrating that plasma IgG from healthy individuals or Crohn’s disease (CD) patients bound via the Fc region to a high molecular weight protein detectable in the mucus granules of goblet cells and mucus layer of the intestines.^25,26^ This suggests a potential role for FCGBP in IgG-dependent regulation of mucus-associated microbes, particularly in the large intestine, and is also consistent with the presence of detectable levels of anti-commensal IgG in colonic mucus of mice.^27^ However, contrary to earlier work, a recent study using purified proteins *in vitro* did not detect any interaction of mouse FCGBP with IgG.^28^ Therefore, the functions of FCGBP in the mucus layer, including the existence and significance of any immune-related function, remain undefined. To date, there is no reported connection between FCGBP and IBD; however, reduced FCGBP has been associated with the onset and progression of colorectal cancer,^29–33^ which can develop secondary to chronic gut inflammation. However, the mechanism underlying this possible relationship also remains to be elucidated.

### Isolation and visualization of mucus-associated bacteria

Most microbiota analyses continue to be performed on fecal bacteria, often due to practical reasons. Relative to mucus-associated samples, fecal samples are easier to collect and enable longitudinal analyses of the microbiota of a single individual. Biopsies of the gut wall can be collected from patients undergoing endoscopy and from experimental mice, but the limited amount of biological material available using this approach presents obvious challenges with the interpretation of the findings. Various technical approaches have been employed to collect and/or analyze mucus-associated bacteria from animal models. These include relatively crude methods such as scraping or vacuum-based suctioning of the mucosa following removal of luminal contents and any visible debris.^6,7,27^ These methods enable the collection of virtually all mucus-associated organisms from the entire mucus layer but are less than ideal for distinguishing inner mucus from outer mucus contents. Since these are terminal procedures, longitudinal analyses obviously cannot be conducted. However, samples that can be collected from any region of the intestine desired and under anaerobic conditions allow for preservation, culture, and isolation of viable, highly oxygen-sensitive organisms for culture and downstream functional analyses including transplants into axenic animals.

Different fractions of luminal microbes in the small versus large intestine are actively coated by IgA or IgG antibodies. This feature has been used extensively to enrich antibody-bound bacteria using flow cytometry before conducting genomic sequencing (IgA-Seq or IgG-Seq)^34^ or functional assays including gnotobiotic transplants. The organisms enriched via these methods include bacteria known to penetrate the mucus layer,^2^ but the point at which they become coated with these antibodies remains unclear, and therefore the utility of this approach for collecting and distinguishing mucus-associated bacteria is unknown. Importantly, whether and how the proximity of commensals to the colonic epithelium impacts homeostatic antibody responses are not very well understood. However, considering that certain commensals can utilize similar mechanisms as pathogens to gain entry into the mucus, it is possible to elicit highly specific responses to non-surface protein antigens generated after microbial engulfment and processing and presentation of lymphocyte receptor epitopes by antigen-presenting cells.

Several technical advances have enabled the visualization of microbes in whole tissue sections that preserve the host-microbiota spatial relationship.^35,36^ These approaches utilize fixed tissue and therefore do not allow for culture and isolation of viable organisms. A commonly utilized approach to examining the spatial segregation of gut bacteria and the mucus layers is bacterial fluorescence in situ hybridization (FISH). With this technique, undisturbed colonic tissues, still containing the luminal contents, are treated with specialized fixatives that preserve the mucus layer in its native form prior to being embedded in paraffin. Transverse tissue sections are then labeled using fluorescent probes that recognize conserved or species-specific bacterial DNA sequences.^37–39^ Although the mucus and microbiota can be effectively visualized, the common fixatives do not efficiently preserve the host tissue for immunostaining analysis. A recently described approach utilizing a specialized fixative formulation was demonstrated to overcome this concern.^40^

A more refined technique that has been utilized to study mucus-associated communities of both humans and rodents involves laser-capture microdissection (LCM) of defined areas of healthy tissue or disease lesions.^41–43^ This approach enables the precise collection of low biomass microbial samples from defined areas of the inner or outer mucus layer or even the epithelium. LCM requires equipment and technical expertise not readily accessible to all investigators, and given the spatial biological diversity along the length of the intestine, samples collected in this way can only be interpreted as representative of the specific area from which they were sourced.

### Studying the interaction of the host and the mucus-associated microbiota

Mechanistic analysis of the functions of specific microbes often necessitates *in vivo* experiments that prove Koch’s postulate that the specific phenotype be reproduced when a pure culture of the organism is inoculated in a susceptible host. In the case of commensals, this generally requires that all other organisms be eliminated from the experimental system. The increased availability of gnotobiotic facilities has allowed for experiments whereby transplantation of pure cultures of known mucus-associated bacteria into axenic or ‘germ-free’ animals enable direct demonstration of specific functions *in vivo*. Though informative when successful, such experiments present important challenges regarding their execution and interpretation.^44^ Many mucus-associated bacteria are strict anaerobes, and colonization can be limited by the oxygen tension of the germ-free intestines. In addition, germ-free mice feature thinner mucus layers and under-developed immune systems, both of which are critical to the interactions of the specific microbes with the host under normal circumstances. Finally, goblet cell-associated antigen passages (GAPS) in the proximal colon, the closure of which is regulated by microbial density prior to weaning,^45^ are likely to be accessible in adult germ-free mice, potentially allowing for aberrant transfer of microbial antigens.

An increasingly prevalent approach to *ex vivo* study of the interaction of the host with the mucus-associated microbiota involves the use of colonic stem cells to generate epithelial monolayers that recapitulate the lineage diversity of the gut epithelium, including mucus-secreting goblet cells.^46–48^ This eliminates the need for cell lines, which may not always be representative of gut epithelial cell biology. More importantly, this approach allows for the differentiation of stem cells from specific regions of the intestine into mature lineages that recapitulate the transcriptional profile of their *in vivo* counterparts. In recent years, various highly innovative systems have been engineered to reproduce the environmental conditions that enable survival of oxygen-consuming host cells without compromising the viability of highly oxygen-sensitive microbes.^49,50^ The merger of such reductionist approaches should facilitate mechanistic discovery going forward, though with the caveat that the mammalian physiology that directly impacts gut function cannot be fully recapitulated *in vitro*.

### Mechanisms of interaction between bacteria and mucus

A well-characterized example of a non-pathogenic interaction between host epithelial cells and commensals occurs in the mouse terminal ileum where *Candidatus arthromitus*, commonly known as segmented filamentous bacteria (SFB), breach the mucus layer and bind directly to epithelial cells.^51^ Indeed, the entire small intestine, including the ileum, is overlaid by a single, looser, and unattached mucus layer, which may potentially make it more permissive to invasion by SFB than the mucus layer of the large intestine. This might explain the absence, to date, of direct evidence of this type of invasion and interaction by non-pathogenic organisms anywhere else along the GI tract, including the colon.

Despite the relatively more robust mucus barrier, the colon mucus harbors many more microbes than the small intestine and certain microbes even gain access to the dense inner mucus layer. The diversity of organisms in the outer mucus layer is highest in regions closest to the epithelium,^41^ further increasing the likelihood of inner layer colonization and a consequent increase in the number of potential antigenic hits on the immune system. The probability of mucus colonization in the large bowel is further enhanced by the combination of slower transit times, increased availability of host-inaccessible carbohydrates left over from small bowel digestion, and the widespread capability to bind to and/or forage mucin O-glycans. Moreover, colonic crypts of both mice and humans harbor a diverse microbiota comprised both aerobic and anaerobic organisms that can also be found in the mucus layers.^52,53^ In addition to *Bacteroidetes* and *Firmicutes*, this crypt-specific core microbiota (CSCM) involves large proportions of *Proteobacteria*, especially in the murine cecum and proximal colon. The growth and expansion of *Proteobacteria* is enhanced by nutrients released from apoptotic epithelial cells,^54^ demonstrating that host-derived factors also facilitate microbial habitation of these privileged mucus-protected sites within the intestines.

Mucins can also serve as an anchor for bacterial colonization of the GI tract. Several taxa, including multiple species of *Lactobacillus* express adhesins that mediate binding to mucin glycans^55^ to facilitate colonization of and motility within, the mucus layer. An estimated 30% of gut commensals express type IV pili (T4P) – long and thin-surface appendages that facilitate bacterial adhesion but are also involved in motility, DNA exchange, and protein uptake.^56^ *Fusobacterium nucleatum* binds to colonic Muc2 and secretes outer membrane vesicles (OMVs) that permeate the mucus layer and can stimulate the gut epithelium.^57^ The genome of *Bacteroides thetaiotaomicron* (*B. theta*) consists of multiple polysaccharide utilization loci but also releases OMVs into the mucus layer that reach the immune system in the lamina propria in a sulfatase-dependent manner^58^ (Figure 1B).

A large fraction of the overall gut microbiota can directly target specific monosaccharides in secreted mucin *O*-glycans which can serve as a source of carbon and nitrogen for commensal organisms. One of the first and best described of these mucin-degraders is the aptly named *Akkermansia muciniphila*.^59^ It has become increasingly clear that the vast majority of commensals are capable of either cleaving or catabolizing at least one of the major mucin *O*-glycan monosaccharides – L-fucose (Fuc), D-galactose (Gal), N-acetyl-D-galactosamine (GalNAc), N-acetyl-D-glucosamine (GlcNAc), and N-acetyl-neuraminic acid (Neu5Ac).^4,60^ Some organisms, referred to as mucin generalists, and including several species of *Muribaculaceae* and *Lachnospiraceae*, can forage all five monosaccharides.^61^ Whether generalists or single monosaccharide foragers, the distinct preferences offer competitive and functional advantages within the gut microenvironment. For example, a recently described consortium of 5-sialic acid and N-acetylglucosamine utilizers can prevent colonization by the nosocomial pathogen *Clostridium difficile*.^61^

The inner mucus layer of the human and mouse colon has consistently been shown to be impermeable to bacteria-sized beads,^38,62^ demonstrating the reduced likelihood of passive encroachment of the gut epithelium by bacteria. However, several species of known mucus-associated bacteria encode multiple motility genes that aid their active movement into and through the mucus layer. This includes genes that encode flagellin monomers, proteins involved in flagellar biosynthesis, and the dozens of proteins that comprise the rotary motor. It also includes adhesin genes that enable binding to mucus but also potentially facilitate gliding motion along the mucus scaffold.^63^ Flagella are positioned on the bacterial cell in two major ways that can directly impact the motility mechanism(s) each can employ. In specific rod-shaped cells, one or more flagella can be positioned at or near one or both ends in what is known as a ‘polar’ or subterminal flagellin arrangement, respectively.^63,64^ Other organisms have flagellar filaments positioned along the length of the cell body in what is known as a ‘peritrichous’ distribution.^63^ Several mucus-associated bacteria, particularly *Lachnospiraceae*, express multiple distinct flagellins that exhibit distinct functional properties *in vivo*.^65^ The significance of this diversity is the subject of continued investigation but likely represents additional adaptations to help evade host control mechanisms designed to limit motility.

### Establishing immune homeostasis with the mucus-associated microbiota

#### Innate sensing of bacterial flagellin

The existence of bacteria in such proximity to the epithelium triggers immunoregulatory programs that prevent activation of the immune system. Flagellated bacteria belonging to the families *Lachnospiraceae* and *Ruminococcaceae* are enriched in transverse folds of the mouse proximal colon.^43^ Accordingly, Toll-like receptor 5 (TLR5), the receptor for flagellin is constitutively expressed by epithelial cells in the proximal colon (Figure 1B) as well as Paneth cells in the small intestine.^66^ In neonates, TLR5 is expressed on epithelial cells in both the small and large intestines and regulates colonization by flagellated bacteria with important ramifications for microbiota composition into adulthood.^66,67^ After weaning, expression in the small intestine is downregulated in all but Paneth cells.^66^ Organoids generated *in vitro* from proximal colonic crypt stem cells also stably express TLR5 arguing that neither microbial nor immune signals are necessary to induce TLR5 expression.^66^ In epithelial cells, the NOD-like receptor (NLR) family caspase recruitment domain-containing protein 4 (NLRC4) can also be activated downstream of flagellin-sensing by NLR family apoptosis inhibitory proteins (NAIPs) in response to intracellular replication of pathogenic bacteria, such as *Salmonella typhimurium*. This results in the death of epithelial cells and expulsion into the gut lumen.^68^ However, this pathway appears dispensable for flagellin-induced gene transcription in the colonic epithelium.^66^ Instead, the ability to detect and respond to commensal-derived flagellin via TLR5 throughout life appears to be an intrinsic feature of epithelial cells and part of the genetic program of epithelial cell precursors and the mature enterocytes into which they differentiate. This is consistent with the non-redundant role of TLR5 as an intermediary in the generation of anti-flagellin antibody responses that regulate the composition and motility of the colonic microbiota.^69^ Considering the diversity of flagellins expressed even in phylogenetically related mucus-associated bacteria, evasion of TLR5-mediated sensing by epithelial cells is likely. Thus, in addition to epithelial cells, flagellin can also potently activate the NAIP/NLRC4 pathway in mouse and human macrophages.^70–73^ NAIP/NLRC4 in colonic macrophages likely represent a mechanism for regulating responses to such flagellated bacteria or flagellin antigens that cross the epithelial barrier.

#### Anti-commensal antibodies in control of microbial colonization and activity

The presence of immunoglobulins in the intestinal lumen provides an added layer of defense against epithelial invasion by pathogenic and commensal microbes. Intestinal IgM, as well as class-switched IgA and IgG antibodies, figure prominently in host–microbiota interactions from birth and extend throughout life. IgA is by far the most abundant immunoglobulin isotype present in the intestines and is involved in both exclusion of, and colonization by, mucus-associated commensals (Figure 1B). *Bacteroides fragilis* has evolved mechanisms for utilizing IgA to facilitate its engraftment within the intestines,^74,75^ and members of the phylum Firmicutes, especially those in the *Lachnospiraceae* family, are significantly decreased in patients with selective IgA deficiency.^76^ The protective role of intestinal IgG antibodies has also been widely studied but predominantly in the context of enteric infection.^77^ However, several recent studies (discussed below) offer compelling evidence of the involvement of anti-commensal IgG in promoting homeostasis in both the neonatal and adult stages of life.

IgA and IgG antibodies can be generated by either T-dependent or T-independent mechanisms, meaning, with or without the help of CD4 T cells, respectively. In the latter case, this occurs most commonly via direct stimulation of B cells via innate pattern-recognition receptors. T-independent anti-commensal antibodies generally target conserved carbohydrate moieties present in diverse bacteria ^78^ and, because of this, are sometimes referred to as being polyreactive. It should be noted that in this context, the term polyreactive relates to the diversity of microbes that can be recognized based on expression of common moieties, and not that these antibodies can bind a multiplicity of distinct antigens. In contrast, T-dependent antibodies target-specific protein epitopes either expressed externally or displayed by professional antigen-presenting cells after engulfment and degradation of microbes or microbial components. The predominant antibody isotype produced in the mammalian intestine and thus targeting gut bacteria is IgA. Rodents express a single isoform of IgA making it impossible to determine whether an IgA molecule from a wild-type mouse was produced in a T-dependent or T-independent manner. Therefore, in experimental settings, the distinction is based on whether the response can be induced when T-dependent antibody production is missing or impaired, for example, in T cell-deficient mice. Humans, on the other hand, produce two IgA subtypes, with IgA1 believed to be predominantly T-dependent and IgA2, T-independent.^79,80^ In contrast, both humans and mice express four distinct subtypes of IgG (IgG1-4 in humans, and IgG1, IgG2a/c, IgG2b, and IgG3 in mice). In both species, the generation of IgG1 is largely T-dependent, whereas IgG2 in humans and IgG3 in mice are generated in a T-independent manner and mainly target bacterial carbohydrate epitopes.^81^

The role of T-independent anti-commensal antibodies in establishing and maintaining microbial tolerance throughout life has been extensively demonstrated, especially in recent years. These include maternally derived IgA and IgG antibodies that limit immunoreactivity to the microbiota in early life,^82^ IgA antibodies that actively sequester the luminal bacteria in the small and large intestines,^1^ as well as anti-murein lipoprotein (MLP) IgG antibodies that target Gram-negative commensals and cross-react with MLP-expressing pathogens to limit systemic infection.^83^ Interestingly, polyreactive IgA produced by colonic plasma cells did not seem to target mucus-associated bacteria including firmicutes in the colon, further supporting the notion that at least some mucus-associated microbes necessitate high-affinity T-dependent antibody-mediated control.^84^

T-dependent antibody responses are known to be evoked by enteric pathogens – which also invade the mucus layer to gain access to the epithelium – and are both proportional to the threat sensed by the host and often necessary for effective clearance of the pathogen. However, the role of T-dependent antibodies in controlling commensal organisms is less well defined, with few examples of such interactions reported. IgA coating of luminal bacteria in both the small and large intestines was largely independent of T cells, except for SFB and *Mucispirillum*, both of which penetrate the mucus layer. Interestingly, the seemingly T-dependent coating of both organisms was also independent of somatic hypermutation Tfh cells,^1^ the latter of which can be directly stimulated to aid in the production of anti-commensal IgA.^85^ These studies provide evidence of the role of T-dependent IgA in control of a subset of gut microbes that can penetrate the mucus layer. Interestingly, total IgA-coated organisms isolated from IBD patient feces include at least a subset of organisms that efficiently colonize the colonic mucus layer and increase susceptibility of colonized mice to experimental colitis.^2^ The IgA-coated bacteria, which represent a minority of the total luminal community, are coated by both IgA1 and IgA2^86^ suggesting potential cooperation between T-dependent and T-independent responses in luminal sequestration.

**Figure 2.**
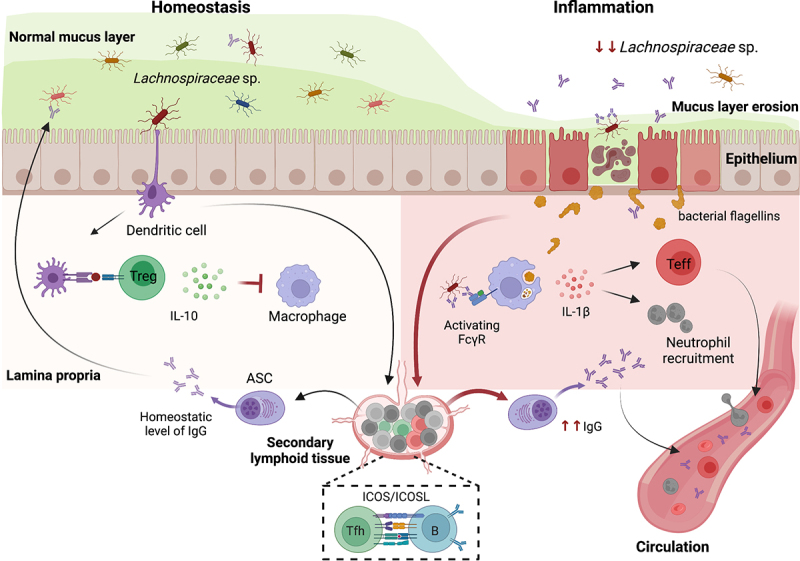
Examples of the crosstalk between mucus-associated bacteria and the intestinal immune system in health and disease. Under homeostatic conditions, the peaceful existence in the mucus layer of bacteria, including members of the *Lachnospiraceae* and *Ruminococcaceae* families, is aided by coordinated antigen collection by lamina propria myeloid cells including dendritic cells and macrophages. Intestinal dendritic cells promote the differentiation and/or maintenance of IL-10-producing Treg cells. IL-10 inhibits commensal-dependent activation of lamina propria macrophages, maintain the macrophages in a quiescent state. In addition, the presentation of commensal-derived antigens by B cells in the gut-draining lymph nodes induces differentiation into IgG+ antibody-secreting cells (ASCs) with the aid of ICOS-ICOSL-dependent T-follicular helper (Tfh) cells. This T-dependent ASC-derived IgG crosses the epithelium into the mucus layer and helps regulate the resident microbial communities. In contrast, the inflammatory state is characterized by an erosion of the mucus layer resulting in increased availability and opsonization of commensal antigens. This enables dysregulated differentiation and expansion of pro-inflammatory/effector CD4 T cells as well as hyperproduction of IgG antibodies to antigens including *Lachnospiraceae* flagellins. Excessive activation of intestinal macrophages via the interaction of IgG immune complexes with activating Fc gamma receptors (FcγR) induces a pro-inflammatory phenotype including secretion of cytokines such as IL-1β which can promote neutrophil recruitment. In CD, the chronic inflammatory state is also characterized by an overall reduction in the abundance of *Lachnospiraceae* bacteria and a breach in the so-called ‘mucosal firewall’ resulting in effector CD4 T cells and anti-commensal IgG antibodies entering the systemic circulation.

T-dependent IgG also appears to be involved in the regulation of host-microbiota crosstalk at the mucosal border as *Akkermansia muciniphila*, which can mount appreciable effector cell responses under specific conditions, also induces a robust T-dependent IgG1 response in mice under homeostatic conditions.^87^ Furthermore, mice with defective production of T-dependent antibodies (ICOSL-deficient mice) harbor reduced IgA and IgG antibodies specific to mucosal antigens, including antigens derived from multiple isolates of known mucus-associated bacteria^27^ (Figure 2). The potential colitogenicity of the microbiota appears to increase in the absence of this type of antibody-mediated control as ICOSL-deficiency predisposes to the development of severe, rapid onset colitis when immune regulation is genetically or transiently perturbed.^27^

#### Microbial induction of T cell-mediated immune regulation

From the earliest stages of life, mucosal antigens are actively involved in promoting the development and expansion of a robust arsenal of regulatory T cells (Treg cells) that provide beneficial suppression of anti-commensal immunity well into adulthood. Mucus-producing goblet cells serve as conduits for Treg-inducing mucosal antigens during a critical window during the postnatal period.^45^ Although the specific antigens driving this differentiation and expansion have not been clearly defined, the time interval of postnatal days 11–20 in mice is associated with the rapid and organized acquisition of diverse organisms via maternal and potentially environmental transfer. Further, the transfer of these antigens via goblet cells suggests they derive from organisms that intimately interact with the mucus layer from the earliest stages of colonization. Experiments in which specific microbes were transferred into adolescent or adult germ-free mice have demonstrated the potential of many different mucus-associated bacteria or bacterial consortia leading to induction and/or accumulation of intestinal regulatory T cells. These organisms include *Bacteroides fragilis*,^88^ *Helicobacter* spp.,^89,90^ the *Lachnospiraceae* family member *Roseburia intestinalis*,^65^ and a consortium including several species of *Lachnospiraceae*.^91^

A key T cell-derived mediator of gut immune homeostasis is the immunoregulatory cytokine interleukin-10 (IL-10) which, in the intestines, is necessary to actively limit macrophage activation in response to commensal-derived triggers.^92^ Mucus-associated bacteria, including *Bacteroides fragilis, B. acidifaciens, Akkermansia muciniphila*, and *Bifidobacterium* spp., either induce T cell expression of IL-10 or require IL-10 to limit their activation of the immune system.^93–96^ It is likely that all these organisms utilize distinct mechanisms to trigger IL-10 production, but the activation of this pathway by such phylogenetically distinct organisms suggest that CD4 T cell production of IL-10 is also a dominant mechanism for tolerance of specifically mucus-associated bacteria. Expression of IL-10 by CD4 T cells increases significantly in the colonic lamina propria of mice in which production of T-dependent antibodies is impaired. Accordingly, simultaneous ablation of T-dependent antibodies and CD4 T cell-derived IL-10 in either early life or adulthood, results in rapid onset of colonic inflammation.^27^ These findings, coupled with the fact that *Akkermansia muciniphila* induces both IgG1 and IL-10, support the theory that specific mucus-associated bacteria trigger T-dependent antibodies and CD4 T cell-derived IL-10, and that synergy between both pathways may be critical for preventing pro-inflammatory responses to these microbes. Interestingly, prior to the onset of colitis that typically occurs in IL-10-deficient mice, the inner mucus layer was found to be significantly thicker but more permeable to bacteria-sized beads.^62^ This suggests a potential role for IL-10 might IL-10 in controlling mucus production and/or mucus layer integrity, further regulating access to the epithelium by mucus-associated bacteria.

### Mucus-associated bacteria and gastrointestinal disease

Due to the non-redundant protective function of the gut mucus layer, factors that either directly or indirectly promote erosion or penetration of this layer can induce significant disturbances in microbiota composition, creating favorable environments for bacterial invasion and onset of gut inflammation. For example, the absence of fiber in the diet deprives commensals of a reliable source of nutrients, which results in excessive foraging of the mucus layer. This leads to microbiota dysbiosis, which favors outgrowth of pathogenic organisms and enhances susceptibility to colitis.^97^ Dietary emulsifiers, common components of processed foods, also promote bacterial encroachment of the colonic epithelium, which favors the development of metabolic syndrome and susceptibility to, or severity of, experimental colitis.^98,99^ It is reasonable to speculate that these observations are due to the direct effects of emulsifiers on the structure and function of mucus. However, current experimental evidence suggests that emulsifiers license pathobionts such as adherent-invasive *E. coli* (AIEC) for mucus penetration and epithelial activation by stimulating upregulation of virulence and motility factors.^100,101^ Interestingly, under these conditions, mucin expression by colonic GCs is upregulated in response to AIEC.^100^ Such enhanced mucus secretion is a typical response to epithelial encroachment and is seen in the presence of enteric infections by helminths, protozoan parasites, and pathogenic bacteria.^102^ This response can further disrupt the mucosal ecosystem and further exacerbate pathogen-induced dysbiosis leading to inflammation.

#### Pathobionts and IBD

The etiology of IBD is believed to involve both the overgrowth of harmful opportunists or pathobionts and the loss of beneficial bacteria. The possible relationship between CD and mucus is still somewhat controversial, whereas it is established and widely accepted that UC patients display a thinner mucus layer, reduced GCs, and defective mucus production and secretion. In the quest to understand the role of specific bacteria in promoting gastrointestinal disorders including IBD, correlative as well as mechanistic associations have been made between specific mucus-associated bacteria and chronic gut inflammation. In mice colonized with the Gram-negative mucus-associated pathobiont *Mucispirillum schaedleri*, combined deficiency of IBD-susceptibility gene nucleotide-binding oligomerization domain-containing protein 2 (*Nod2*) and the cytochrome b-245 beta-chain subunit of phagocyte NADPH oxidase develop spontaneous colitis after weaning as the protection afforded by maternal antibodies begins to wane.^103^ The disease is characterized by an expansion of *M. schaedleri* due to impaired recruitment of luminal neutrophils necessary to clear the bacteria. This illustrates how a defect in innate immune-mediated regulation can enable mucus-associated bacterial overgrowth, which overwhelms the host's adaptive immunity to promote colonic inflammation.

*Ruminococcus gnavus* (*R. gnavus*) is also significantly increased in both CD and UC, sometimes transiently reaching a relative abundance of greater than 50% of patient fecal bacteria and dominated by strains capable of surviving the oxidative stressors characteristic of the inflamed gut.^43,104,105^ Therefore, like AIEC,^106^ the expansion of *R. gnavus* in IBD is possibly the response of an opportunist to local conditions. Notably, however, patient isolates of *R. gnavus* can be distinguished by the presence or absence of a thick polysaccharide capsule that masks the cell wall components. Strains expressing this capsule are largely inert and favor tolerogenic responses *in vivo*. In contrast, unencapsulated strains enhance the severity of inflammation,^107,108^ supporting the involvement of *R. gnavus* as a microbial driver of IBD.

#### Inverse correlations of mucus-associated bacteria and colonic inflammation

Associations have also been made between low abundance, albeit in the feces, of known mucus-associated bacteria and disease activity of severity in IBD.^109^ Reduced fecal and mucosal abundance of *Faecalibacterium prausnitzii* has been observed in the feces of patients with CD and UC.^110–112^ The relative abundance of members of the families *Ruminococcaceae* and *Lachnospiraceae*, including *Roseburia hominis* are also negatively correlated with disease activity in CD and UC.^113–116^ *Lachnospiraceae* were among the most decreased bacterial families in the ileocecal biopsies in treatment-naïve Crohn’s disease patients,^117^ suggesting that the elimination or suppression of this family of organisms potentially contributes to disease onset. In support of this theory, transplantation of a randomly selected consortium of 23 *Lachnospiraceae* organisms enriched from the mouse feces, cecal contents, and cecal tissue was able to repair the dysbiosis observed in NLRP12 inflammasome-deficient mice and limit the severity of the colitis that ensued when the mice were subsequently administered dextran sulfate sodium (DSS).^118^

#### Anti-Lachnospiraceae immune responses in IBD

The total levels of fecal IgG are non-specifically elevated and bind luminal microbes in both UC and CD.^119^ However, immunoreactivity of antigens derived specifically from members of the *Lachnospiraceae* family are strongly implicated in IBD. Specifically, reactivity of *Lachnospiraceae* flagellin antigens continues to be utilized as a diagnostic tool in IBD and IgG seroreactivity for CBir1, a flagellin antigen isolated from cecal *Lachnospiraceae*, is significantly increased in patients with CD, but not in UC patients or healthy controls. This reactivity is present in CD cohorts collected from different locations and in different age groups, making it an effective biomarker for CD diagnosis and prognosis.^120–122^ Alexander et al. recently expanded the spectrum of flagellin antigens that are targeted by CD serum IgG to an array of human and mouse *Lachnospiraceae* isolates and observed that approximately 30% of CD patients displayed an elevated serum IgG response to more than 10 different flagellins. This multi-flagellin reactivity was highly correlated with heightened responses to CBir1 flagellin, but not with reactivities to flagellins from *Salmonella dublin* (*S. dublin*) or *E. coli*. In further support of a highly targeted response to specific commensal-derived antigenic epitopes, these patients concomitantly displayed increased flagellin-specific CD4+ effector/memory cells in the circulation ^123^ (Figure 2). Within this extremely diverse family, many of the organisms can express multiple flagellins, adding an additional layer of molecular complexity that likely impacts recognition by the host immune system. At a minimum, the observation that the targeted deletion of *Lachnospiraceae* flagellin-specific CD4 T cells in the circulation can prevent experimental colitis in mice ^124^ makes this axis a potentially viable target for intervention in CD.

Despite these robust IgG responses, it is still unknown whether the antibodies detectable in the circulation of CD patients were induced by disseminated bacteria, produced by circulating B cells that originate in the gut, or produced in the gut before entering the circulation. Thus, it remains to be determined whether these well-established anti-*Lachnospiraceae* responses are a cause or consequence of disease. IgG, acting via activating Fc gamma receptors (FcγRs) on gut-resident macrophages, potently drives inflammation in UC.^125,126^ Accordingly, a single-nucleotide polymorphism in *FCGR2A* that reduces the affinity of this receptor for IgG is protective in UC^127,128^ supporting the notion that massive IgG response to known mucus-associated organisms in CD could indeed be a contributor to disease induction and/or progression (Figure 2). On the other hand, a similar magnitude of *Lachnospiraceae* flagellin-specific serum IgG response was observed in healthy children at 12–24 months of age. This response likely originated in the infant gut and was not passively acquired since comparable responses were not detected in mothers or unrelated adult control subjects.^129^ This period in humans also coincides with the introduction of solid food and a turbulent period of microbiota diversification and expansion that drives the immunological “weaning reaction” documented in mice.^130^ Interestingly, the heightened response eventually receded to the level of healthy adults by 7 years of age.^129^ Thus, an alternative possibility is that the increased IgG antibodies targeting *Lachnospiraceae* flagellins found in adult CD patients result from an over-exuberant recall response toward mucus-associated commensal antigens triggered or enhanced by the co-occurrence of other disease-promoting events.

## Conclusions and future perspectives

The colon mucus layer represents an ecosystem that is vital to the host-microbial relationships essential to mammalian life. There is still much to be learned regarding the host adaptations that facilitate this co-existence and whether and how those responses are related to the etiology and/or progression of chronic intestinal inflammation. Our understanding will improve following investigations into the nature of the physical and biochemical interactions that define the mutualism between mucus-associated microbes and the host, the potential involvement of such organisms in the initiation and/or perpetuation of gut inflammation, and the potential utility of mucus-associated microbes or microbial products toward therapeutic ends. Several emerging technical and technological advances point to an exciting future of discovery regarding host-microbiota mutualism at the colonic mucosal border. For example, continued refinement of technical approaches for (1) aseptic collection of microbes directly from the mucus layers of the colon, and (2) efficient characterization of the gene expression patterns at specific points should enable determination of the microbial molecular pathways that promote existence of specific organisms in this locale, including direct and indirect communication with the immune system. Additional improvements in, and availability of, approaches for high-throughput culture and isolation of distinct organisms from these delicate biological samples will lead to mechanistic studies at the level of bacterial strains. The differential manipulability of specific organisms implicated in gastrointestinal disease remains a persistent challenge. Genetic modifications of certain Protobacteria and Bacteroides have provided important insight into the potential functions of organisms including *E. coli*, and *B. theta*, and *B. fragilis*. However, other organisms, many of which still have not been cultured in a laboratory, are less genetically tractable and for now, this limits our ability to study their function. Recent innovative advances offer hope for precise genetic manipulation of individual organisms or communities of varying size and diversity.^131–134^ Many techniques deployed to date have predominantly engineered the bacteria *in vitro*, but others have demonstrated successful *in situ* modification of microbes recovered from the lumen of recipient animals.^135,136^ The latter approaches raise the possibility of whole-microbiome engineering *in vivo* for therapeutic purposes, although novel or modified approaches may be necessary to specifically target consequential organisms residing deep within the colonic mucus layer.
